# 4-(4-Methyl­phenyl­sulfon­yl)piperazin-1-ium tri­fluoro­acetate

**DOI:** 10.1107/S1600536813015900

**Published:** 2013-06-19

**Authors:** S. Sreenivasa, N. R. Mohan, T. Madhu Chakrapani Rao, P. A. Suchetan, B. S. Palakshamurthy, Vijithkumar

**Affiliations:** a Department of Studies and Research in Chemistry, Tumkur University, Tumkur, Karnataka 572 103, India; b Tadimety Aromatics Pvt Ltd, Hirehally Industrial Area, Tumkur, Karnataka 572 168 , India; c Department of Studies in Chemistry, U.C.S., Tumkur University, Tumkur, Karnataka 572 103, India; d Department of Studies and Research in Physics, U.C.S., Tumkur University, Tumkur, Karnataka 572 103, India; e Soild State and Structural Chemistry Unit, Indian Institute of Science, Bangalore 560 012, India

## Abstract

In the title salt, C_11_H_17_N_2_O_2_S^+^·CF_3_COO^−^, the cation is protonated at the secondary piperazine N atom. The dihedral angle between the benzene ring and the piperazine mean plane is 85.54 (10)°. In the crystal, cations and anions are connected by two types of strong N—H⋯O hydrogen bonds into chains extending along [101]. The chains are further assembled into (10-1) layers *via* stacking inter­actions between benzene rings of the cations [centroid–centroid distance = 3.7319 (13) Å] and a C—H⋯O inter­action involving a piperazine C—H group and a sulfonyl O atom. Another C—H⋯O inter­action between the piperazine ring and the sulfonyl group connects the ions into a three-dimensional network.

## Related literature

For the synthesis, characterization and biological activity of piperazine derivatives, see: Gan *et al.* (2009*a* ▶,*b* ▶). For hydrogen-bond motifs, see: Bernstein *et al.* (1995 ▶).
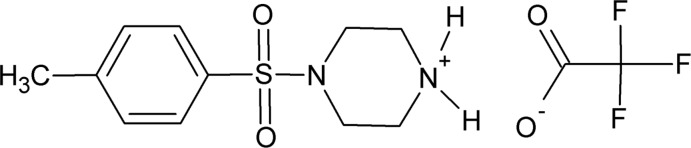


## Experimental

### 

#### Crystal data

C_11_H_17_N_2_O_2_S^+^·C_2_F_3_O_2_^−^
*M* *_r_* = 354.35
Monoclinic, 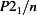

*a* = 7.8796 (6) Å
*b* = 22.5891 (15) Å
*c* = 9.4626 (7) Å
β = 110.446 (3)°
*V* = 1578.2 (2) Å^3^
*Z* = 4
Mo *K*α radiation
μ = 0.26 mm^−1^
*T* = 100 K
0.24 × 0.22 × 0.20 mm

#### Data collection

Bruker APEXII diffractometer
Absorption correction: multi-scan (*SADABS*; Bruker, 2009 ▶) *T*_min_ = 0.941, *T*_max_ = 0.950
11562 measured reflections
2781 independent reflections
2417 reflections with *I* > 2σ(*I*)
*R*_int_ = 0.030

#### Refinement

*R*[*F* ^2^ > 2σ(*F* ^2^)] = 0.037
*wR*(*F* ^2^) = 0.094
*S* = 1.04
2781 reflections
217 parameters
1 restraint
H atoms treated by a mixture of independent and constrained refinement
Δρ_max_ = 0.86 e Å^−3^
Δρ_min_ = −0.55 e Å^−3^

### 

Data collection: *APEX2* (Bruker, 2009 ▶); cell refinement: *APEX2* and *SAINT-Plus* (Bruker, 2009 ▶); data reduction: *SAINT-Plus* and *XPREP* (Bruker, 2009 ▶); program(s) used to solve structure: *SHELXS97* (Sheldrick, 2008 ▶); program(s) used to refine structure: *SHELXL97* (Sheldrick, 2008 ▶); molecular graphics: *Mercury* (Macrae *et al.*, 2008 ▶); software used to prepare material for publication: *SHELXL97*.

## Supplementary Material

Crystal structure: contains datablock(s) I, global. DOI: 10.1107/S1600536813015900/gk2578sup1.cif

Structure factors: contains datablock(s) I. DOI: 10.1107/S1600536813015900/gk2578Isup2.hkl

Supplementary material file. DOI: 10.1107/S1600536813015900/gk2578Isup3.cml

Additional supplementary materials:  crystallographic information; 3D view; checkCIF report

## Figures and Tables

**Table 1:** Hydrogen-bond geometry (Å, °)

*D*—H⋯*A*	*D*—H	H⋯*A*	*D*⋯*A*	*D*—H⋯*A*
N2—H1*N*2⋯O4^i^	0.87 (3)	1.91 (3)	2.782 (2)	174 (2)
C8—H8*B*⋯O1^ii^	0.97	2.45	3.328 (2)	150
C9—H9*B*⋯O2^iii^	0.97	2.43	3.146 (2)	130
N2—H2*N*2⋯O3	0.86 (2)	1.86 (2)	2.690 (2)	163 (2)

Symmetry codes: (i) 
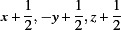
; (ii) 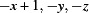; (iii) 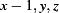.

**Fig. 1.**
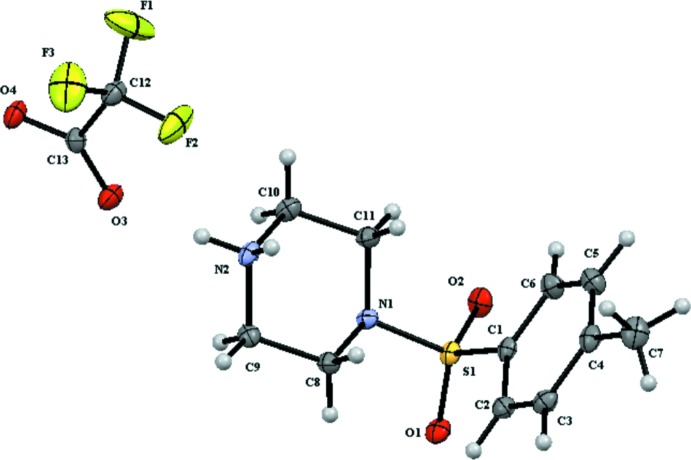
Molecular structure of the title compound, showing the atom-labeling scheme. Displacement ellipsoids are drawn at the 50% probability level.

**Fig. 2.**
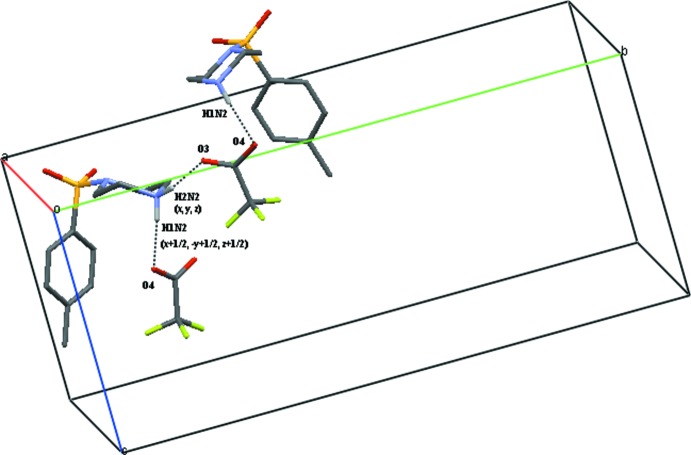
Chain of anions and cations connected via N—H···O hydrogen bonds. The hydrogen bonds are shown as dashed lines and the hydrogen atoms not involved in hydrogen bonds are omitted.

**Fig. 3.**
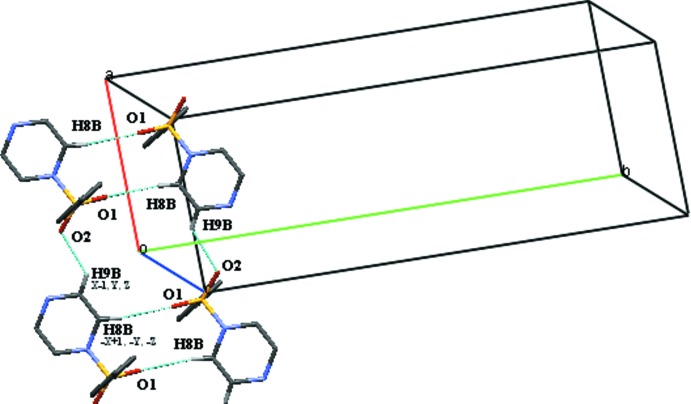
Molecular packing in the title compound displaying R22(10) rings and C(6) chains. Trifluoroacetate ion is omitted for clarity.

**Fig. 4.**
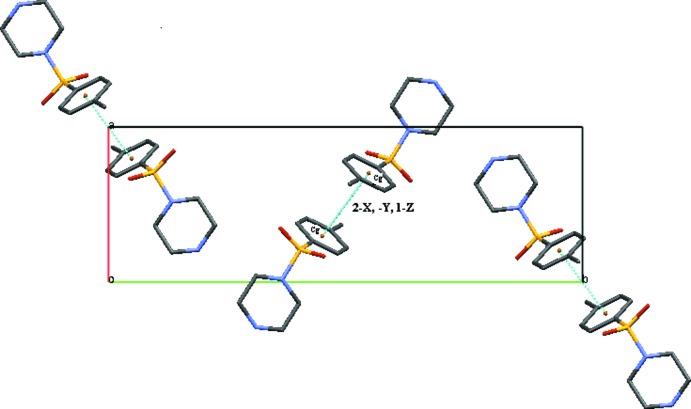
Aromatic π-π stacking interactions observed in the crystal structure.

## supplementary crystallographic information

### Comment

Numerous piperazine derivatives like aryl amide, sulfonamides, Mannich bases, Schiff bases, thiazolidinones, azetidinones, imidazolinones have shown a wide spectrum of biological activities *viz*. anti-inflammatory, antibacterial, antimalarial, anticonvulsant, antipyretic, antitumor, anthelmintics, analgesic, antidepressant, antifungal, antitubercular, anticancer, antidiabetic (Gan *et al.*, 2009*a*,*b*). Keeping this in mind, we synthesized the title compound and here we report its crystal structure.

The title molecular salt, C_11_H_17_SO_2_N_2_^+^.CF_3_COO^-^, crystallizes in monoclinic crystal system and *P*2_1_/*n* space group. The cation is protonated at the secondary N atom of the piperazine ring (Fig. 1). The piperazine ring adopts a chair conformation and the dihedral angle between the benzene ring and the piperazine ring (considering the mean plane formed by all the non-hydrogen atoms) in the cation is 85.54 (10)*^o^*. In the crystal, the ions are connected by strong N2—H1(N2)···O4 and N2—H2(N2)···O3 hydrogen bonds (Fig. 2, Table 1). The cations are further connected through weak C8—H8B···O1 and C9—H9B···O2 interactions forming chain C(6) and ring *R*_2_^2^(8) motifs (Bernstein *et al.*1995) (Fig. 3, Table 1). The crystal structure is further stabilized by aromatic π-π stacking interactions.

### Experimental

A mixture of *tert*-butyl 4-[(4-methylphenyl)sulfonyl]piperazine-1-carboxylate (0.002 moles, 1 gram) (1), trifluroacetic acid (TFA) (0.013 moles, 1 ml) in 1,2-dichloroethane (10 ml) was refluxed for 2 h. Reaction mixture was then cooled to room temperature and concentrated to get crude pale yellow colored solid 1-[(4-methylphenyl)sulfonyl]piperazine (2). The crude compound (2) was purified by column chromatography using petroleum ether:/ethyl acetate (7:3) as eluent to get white coloured solid (melting point = 518 K), which was further recrystallized from petroleum ether/ dichloromethane (1:1) to obtain colorless crystals suitable for diffraction studies.

### Refinement

The hydrogen atoms attached to N were located in difference maps. The distance H1N2-N2 was restrained to 0.86 (2) Å whereas H2N2 was freely refined. The remaining H atoms were positioned with idealized geometry using a riding model with C—H = 0.93 - 0.97 Å. The isotropic displacement parameters for all H atoms were set to 1.2 times *U*_eq_ of the parent atom or 1.5 times that of the parent atom for CH_3 _group.

### Crystal data

**Table:**

C_11_H_17_N_2_O_2_S^+^·C_2_F_3_O_2_^−^	prism
*M**_r_* = 354.35	*D*_x_ = 1.491 Mg m^−^^3^
Monoclinic, *P*2_1_/*n*	Melting point: 518 K
Hall symbol: -P 2yn	Mo *K*α radiation, λ = 0.71073 Å
*a* = 7.8796 (6) Å	Cell parameters from 2417 reflections
*b* = 22.5891 (15) Å	θ = 1.8–25.0°
*c* = 9.4626 (7) Å	µ = 0.26 mm^−^^1^
β = 110.446 (3)°	*T* = 100 K
*V* = 1578.2 (2) Å^3^	Prism, colourless
*Z* = 4	0.24 × 0.22 × 0.20 mm
*F*(000) = 736	

### Data collection

**Table:**

Bruker APEXII diffractometer	2781 independent reflections
Radiation source: fine-focus sealed tube	2417 reflections with *I* > 2σ(*I*)
Graphite monochromator	*R*_int_ = 0.030
Detector resolution: 1.03 pixels mm^-1^	θ_max_ = 25.0°, θ_min_ = 1.8°
phi and ω scans	*h* = −9→9
Absorption correction: multi-scan (*SADABS*; Bruker, 2009)	*k* = −26→24
*T*_min_ = 0.941, *T*_max_ = 0.950	*l* = −11→10
11562 measured reflections	

### Refinement

**Table:**

Refinement on *F*^2^	Primary atom site location: structure-invariant direct methods
Least-squares matrix: full	Secondary atom site location: difference Fourier map
*R*[*F*^2^ > 2σ(*F*^2^)] = 0.037	Hydrogen site location: inferred from neighbouring sites
*wR*(*F*^2^) = 0.094	H atoms treated by a mixture of independent and constrained refinement
*S* = 1.04	*w* = 1/[σ^2^(*F*_o_^2^) + (0.042*P*)^2^ + 1.4486*P*] where *P* = (*F*_o_^2^ + 2*F*_c_^2^)/3
2781 reflections	(Δ/σ)_max_ = 0.001
217 parameters	Δρ_max_ = 0.86 e Å^−^^3^
1 restraint	Δρ_min_ = −0.55 e Å^−^^3^
0 constraints	

### Special details

**Table:**

Geometry. All e.s.d.'s (except the e.s.d. in the dihedral angle between two l.s. planes) are estimated using the full covariance matrix. The cell e.s.d.'s are taken into account individually in the estimation of e.s.d.'s in distances, angles and torsion angles; correlations between e.s.d.'s in cell parameters are only used when they are defined by crystal symmetry. An approximate (isotropic) treatment of cell e.s.d.'s is used for estimating e.s.d.'s involving l.s. planes.
Refinement. Refinement of *F*^2^ against ALL reflections. The weighted *R*-factor *wR* and goodness of fit *S* are based on *F*^2^, conventional *R*-factors *R* are based on *F*, with *F* set to zero for negative *F*^2^. The threshold expression of *F*^2^ > σ(*F*^2^) is used only for calculating *R*-factors(gt) *etc*. and is not relevant to the choice of reflections for refinement. *R*-factors based on *F*^2^ are statistically about twice as large as those based on *F*, and *R*- factors based on ALL data will be even larger.

### Fractional atomic coordinates and isotropic or equivalent isotropic displacement parameters (Å^2^)

**Table:**

	*x*	*y*	*z*	*U*_iso_*/*U*_eq_	
C1	0.7630 (2)	0.06870 (8)	0.2940 (2)	0.0153 (4)	
C2	0.6839 (3)	0.01633 (9)	0.3194 (2)	0.0183 (4)	
H2	0.6086	−0.0057	0.2388	0.022*	
C3	0.7192 (3)	−0.00242 (9)	0.4665 (2)	0.0196 (4)	
H3	0.6670	−0.0373	0.4841	0.023*	
C4	0.8315 (3)	0.03019 (9)	0.5883 (2)	0.0192 (4)	
C5	0.9097 (3)	0.08191 (9)	0.5598 (2)	0.0214 (4)	
H5	0.9856	0.1038	0.6404	0.026*	
C6	0.8771 (3)	0.10158 (9)	0.4140 (2)	0.0198 (4)	
H6	0.9307	0.1362	0.3967	0.024*	
C7	0.8684 (3)	0.00917 (10)	0.7478 (2)	0.0259 (5)	
H7A	0.9878	0.0212	0.8105	0.039*	
H7B	0.8600	−0.0332	0.7488	0.039*	
H7C	0.7809	0.0261	0.7855	0.039*	
C8	0.3628 (2)	0.09780 (8)	0.0749 (2)	0.0167 (4)	
H8A	0.3811	0.0860	0.1777	0.020*	
H8B	0.3528	0.0623	0.0149	0.020*	
C9	0.1909 (3)	0.13368 (9)	0.0128 (2)	0.0171 (4)	
H9A	0.1691	0.1438	−0.0917	0.020*	
H9B	0.0889	0.1106	0.0168	0.020*	
C10	0.3658 (3)	0.22462 (9)	0.1004 (2)	0.0193 (4)	
H10A	0.3766	0.2598	0.1617	0.023*	
H10B	0.3462	0.2371	−0.0022	0.023*	
C11	0.5387 (3)	0.18885 (8)	0.1599 (2)	0.0175 (4)	
H11A	0.6391	0.2118	0.1521	0.021*	
H11B	0.5646	0.1794	0.2654	0.021*	
C12	−0.0777 (3)	0.32181 (9)	0.1546 (2)	0.0218 (5)	
C13	−0.1238 (2)	0.30961 (9)	−0.0157 (2)	0.0167 (4)	
F1	0.0057 (3)	0.37256 (7)	0.19613 (16)	0.0589 (5)	
F2	0.0210 (2)	0.27979 (6)	0.24299 (14)	0.0485 (4)	
F3	−0.2294 (2)	0.32421 (8)	0.18855 (15)	0.0523 (4)	
N1	0.5186 (2)	0.13357 (7)	0.07164 (17)	0.0146 (3)	
N2	0.2087 (2)	0.18872 (7)	0.10346 (19)	0.0162 (4)	
O1	0.66600 (18)	0.04597 (6)	0.00690 (15)	0.0202 (3)	
O2	0.84473 (18)	0.13710 (6)	0.10568 (15)	0.0213 (3)	
O3	−0.05244 (19)	0.26533 (6)	−0.04921 (15)	0.0215 (3)	
O4	−0.22722 (19)	0.34645 (6)	−0.10056 (15)	0.0231 (3)	
S1	0.70876 (6)	0.09536 (2)	0.10835 (5)	0.01538 (15)	
H1N2	0.222 (3)	0.1792 (10)	0.196 (3)	0.029 (6)*	
H2N2	0.114 (2)	0.2104 (9)	0.066 (2)	0.021 (6)*	

### Atomic displacement parameters (Å^2^)

**Table:**

	*U*^11^	*U*^22^	*U*^33^	*U*^12^	*U*^13^	*U*^23^
C1	0.0127 (9)	0.0189 (10)	0.0163 (10)	0.0046 (8)	0.0074 (8)	0.0021 (8)
C2	0.0177 (10)	0.0179 (10)	0.0197 (10)	0.0016 (8)	0.0071 (8)	−0.0026 (8)
C3	0.0210 (10)	0.0178 (10)	0.0232 (11)	0.0021 (8)	0.0120 (9)	0.0019 (8)
C4	0.0158 (10)	0.0249 (11)	0.0191 (10)	0.0050 (8)	0.0091 (8)	0.0016 (8)
C5	0.0190 (10)	0.0263 (11)	0.0181 (10)	−0.0031 (8)	0.0053 (8)	−0.0028 (8)
C6	0.0162 (10)	0.0218 (11)	0.0222 (11)	−0.0026 (8)	0.0078 (8)	0.0001 (8)
C7	0.0269 (12)	0.0338 (12)	0.0201 (11)	0.0035 (9)	0.0120 (9)	0.0036 (9)
C8	0.0149 (10)	0.0162 (10)	0.0207 (10)	−0.0021 (8)	0.0084 (8)	−0.0026 (8)
C9	0.0158 (10)	0.0212 (10)	0.0152 (10)	0.0002 (8)	0.0066 (8)	−0.0025 (8)
C10	0.0229 (10)	0.0162 (10)	0.0213 (10)	0.0017 (8)	0.0108 (8)	0.0016 (8)
C11	0.0182 (10)	0.0150 (10)	0.0210 (10)	−0.0004 (8)	0.0089 (8)	−0.0023 (8)
C12	0.0272 (11)	0.0196 (11)	0.0181 (10)	0.0076 (9)	0.0071 (9)	0.0025 (8)
C13	0.0141 (9)	0.0194 (10)	0.0169 (10)	0.0003 (8)	0.0058 (8)	0.0015 (8)
F1	0.0970 (14)	0.0423 (9)	0.0240 (8)	−0.0291 (9)	0.0041 (8)	−0.0078 (6)
F2	0.0775 (11)	0.0456 (9)	0.0158 (7)	0.0377 (8)	0.0080 (7)	0.0068 (6)
F3	0.0497 (9)	0.0871 (12)	0.0294 (8)	0.0198 (9)	0.0253 (7)	0.0013 (8)
N1	0.0143 (8)	0.0145 (8)	0.0172 (8)	0.0000 (6)	0.0080 (7)	−0.0012 (6)
N2	0.0178 (9)	0.0181 (9)	0.0142 (9)	0.0062 (7)	0.0075 (7)	0.0028 (7)
O1	0.0220 (7)	0.0234 (8)	0.0177 (7)	0.0056 (6)	0.0099 (6)	−0.0016 (6)
O2	0.0165 (7)	0.0269 (8)	0.0244 (8)	0.0003 (6)	0.0119 (6)	0.0045 (6)
O3	0.0274 (8)	0.0201 (8)	0.0177 (7)	0.0089 (6)	0.0087 (6)	0.0027 (6)
O4	0.0254 (8)	0.0254 (8)	0.0168 (7)	0.0122 (6)	0.0054 (6)	0.0030 (6)
S1	0.0137 (3)	0.0193 (3)	0.0159 (3)	0.00225 (18)	0.00858 (19)	0.00105 (18)

### Geometric parameters (Å, º)

**Table:**

C1—C6	1.392 (3)	C9—H9B	0.9700
C1—C2	1.397 (3)	C10—N2	1.488 (3)
C1—S1	1.7623 (19)	C10—C11	1.514 (3)
C2—C3	1.387 (3)	C10—H10A	0.9700
C2—H2	0.9300	C10—H10B	0.9700
C3—C4	1.394 (3)	C11—N1	1.480 (2)
C3—H3	0.9300	C11—H11A	0.9700
C4—C5	1.391 (3)	C11—H11B	0.9700
C4—C7	1.510 (3)	C12—F1	1.312 (3)
C5—C6	1.385 (3)	C12—F2	1.323 (2)
C5—H5	0.9300	C12—F3	1.343 (3)
C6—H6	0.9300	C12—C13	1.548 (3)
C7—H7A	0.9600	C13—O3	1.242 (2)
C7—H7B	0.9600	C13—O3	1.242 (2)
C7—H7C	0.9600	C13—O4	1.243 (2)
C8—N1	1.479 (2)	N1—S1	1.6576 (16)
C8—C9	1.510 (3)	N2—H1N2	0.87 (3)
C8—H8A	0.9700	N2—H2N2	0.860 (16)
C8—H8B	0.9700	O1—S1	1.4332 (14)
C9—N2	1.489 (2)	O2—S1	1.4340 (14)
C9—H9A	0.9700		
			
C6—C1—C2	120.75 (18)	N2—C10—H10A	109.6
C6—C1—S1	119.58 (15)	C11—C10—H10A	109.6
C2—C1—S1	119.60 (15)	N2—C10—H10B	109.6
C3—C2—C1	119.12 (18)	C11—C10—H10B	109.6
C3—C2—H2	120.4	H10A—C10—H10B	108.1
C1—C2—H2	120.4	N1—C11—C10	109.58 (15)
C2—C3—C4	121.03 (19)	N1—C11—H11A	109.8
C2—C3—H3	119.5	C10—C11—H11A	109.8
C4—C3—H3	119.5	N1—C11—H11B	109.8
C5—C4—C3	118.68 (18)	C10—C11—H11B	109.8
C5—C4—C7	120.96 (18)	H11A—C11—H11B	108.2
C3—C4—C7	120.36 (19)	F1—C12—F2	108.37 (18)
C6—C5—C4	121.48 (19)	F1—C12—F3	106.73 (18)
C6—C5—H5	119.3	F2—C12—F3	104.68 (17)
C4—C5—H5	119.3	F1—C12—C13	112.23 (17)
C5—C6—C1	118.94 (18)	F2—C12—C13	113.76 (16)
C5—C6—H6	120.5	F3—C12—C13	110.57 (16)
C1—C6—H6	120.5	O3—C13—O4	128.86 (18)
C4—C7—H7A	109.5	O3—C13—O4	128.86 (18)
C4—C7—H7B	109.5	O3—C13—C12	116.54 (16)
H7A—C7—H7B	109.5	O3—C13—C12	116.54 (16)
C4—C7—H7C	109.5	O4—C13—C12	114.60 (17)
H7A—C7—H7C	109.5	C8—N1—C11	112.06 (14)
H7B—C7—H7C	109.5	C8—N1—S1	114.04 (12)
N1—C8—C9	109.67 (15)	C11—N1—S1	114.21 (12)
N1—C8—H8A	109.7	C10—N2—C9	110.79 (15)
C9—C8—H8A	109.7	C10—N2—H1N2	110.0 (15)
N1—C8—H8B	109.7	C9—N2—H1N2	109.2 (16)
C9—C8—H8B	109.7	C10—N2—H2N2	106.9 (15)
H8A—C8—H8B	108.2	C9—N2—H2N2	110.2 (15)
N2—C9—C8	109.43 (15)	H1N2—N2—H2N2	110 (2)
N2—C9—H9A	109.8	O1—S1—O2	120.15 (8)
C8—C9—H9A	109.8	O1—S1—N1	106.33 (8)
N2—C9—H9B	109.8	O2—S1—N1	106.28 (8)
C8—C9—H9B	109.8	O1—S1—C1	108.66 (9)
H9A—C9—H9B	108.2	O2—S1—C1	108.65 (9)
N2—C10—C11	110.41 (16)	N1—S1—C1	105.87 (8)
			
C6—C1—C2—C3	0.6 (3)	C9—C8—N1—C11	58.60 (19)
S1—C1—C2—C3	−176.24 (14)	C9—C8—N1—S1	−169.70 (12)
C1—C2—C3—C4	0.1 (3)	C10—C11—N1—C8	−57.2 (2)
C2—C3—C4—C5	−0.6 (3)	C10—C11—N1—S1	171.14 (12)
C2—C3—C4—C7	179.94 (18)	C11—C10—N2—C9	−58.0 (2)
C3—C4—C5—C6	0.5 (3)	C8—C9—N2—C10	58.8 (2)
C7—C4—C5—C6	179.90 (18)	O4—C13—O3—O3	0.00 (8)
C4—C5—C6—C1	0.2 (3)	C12—C13—O3—O3	0.00 (7)
C2—C1—C6—C5	−0.7 (3)	C8—N1—S1—O1	52.91 (14)
S1—C1—C6—C5	176.09 (15)	C11—N1—S1—O1	−176.43 (13)
N1—C8—C9—N2	−58.22 (19)	C8—N1—S1—O2	−177.97 (13)
N2—C10—C11—N1	56.2 (2)	C11—N1—S1—O2	−47.32 (14)
F1—C12—C13—O3	117.9 (2)	C8—N1—S1—C1	−62.54 (14)
F2—C12—C13—O3	−5.6 (3)	C11—N1—S1—C1	68.11 (14)
F3—C12—C13—O3	−123.07 (19)	C6—C1—S1—O1	153.58 (15)
F1—C12—C13—O3	117.9 (2)	C2—C1—S1—O1	−29.56 (17)
F2—C12—C13—O3	−5.6 (3)	C6—C1—S1—O2	21.24 (18)
F3—C12—C13—O3	−123.07 (19)	C2—C1—S1—O2	−161.90 (14)
F1—C12—C13—O4	−61.4 (2)	C6—C1—S1—N1	−92.56 (16)
F2—C12—C13—O4	175.05 (18)	C2—C1—S1—N1	84.30 (16)
F3—C12—C13—O4	57.6 (2)		

### Hydrogen-bond geometry (Å, º)

**Table:**

*D*—H···*A*	*D*—H	H···*A*	*D*···*A*	*D*—H···*A*
N2—H1*N*2···O4^i^	0.87 (3)	1.91 (3)	2.782 (2)	174 (2)
C8—H8*B*···O1^ii^	0.97	2.45	3.328 (2)	150
C9—H9*B*···O2^iii^	0.97	2.43	3.146 (2)	130
N2—H2*N*2···O3	0.86 (2)	1.86 (2)	2.690 (2)	163 (2)

Symmetry codes: (i) *x*+1/2, −*y*+1/2, *z*+1/2; (ii) −*x*+1, −*y*, −*z*; (iii) *x*−1, *y*, *z*.
